# 20p12.3 deletion is rare cause of syndromic cleft palate: case report and review of literature

**DOI:** 10.1186/s13104-015-1828-y

**Published:** 2016-01-02

**Authors:** Saadia Amasdl, Abdelhafid Natiq, Aziza Sbiti, Maria Zerkaoui, Jaber Lyahyai, Saaid Amzazi, Thomas Liehr, Abdelaziz Sefiani

**Affiliations:** Centre de Génomique Humaine, Faculté de Médecine et de Pharmacie, Université Mohammed V , Rabat, Morocco; Département de Génétique Médicale, Institut National d’Hygiène, Rabat, Morocco; Faculté des Sciences, Université Mohammed V, Rabat, Morocco; Institut de Génétique Humaine, Hôpital Universitaire de Jena, Jena, Germany

**Keywords:** *BMP2*, Cleft palate, FISH, 20p12.3 deletion

## Abstract

**Background:**

Orofacial cleft (OFC) is one of the most common congenital malformations with a global incidence of approximately 1/700 live births. Clinically, OFCs can be syndromic or non-syndromic.

**Case presentation:**

A 5 years old boy admitted for genetic evaluation because of psychomotor delay, failure to thrive, dysmorphic features and cleft palate. Conventional cytogenetic showed a notably short p arm of one chromosome 20. FISH analysis identified the derivative chromosome 20 as a de novo 20p12.3 deletion.

**Conclusion:**

We present in this paper a Moroccan patient with syndromic cleft palate caused by a de novo 20p12.3 deletion, and we highlight the interest of FISH in the diagnosis confirmation of chromosomal rearrangement. In practice, 20p12.3 deletion should be considered as an etiological diagnosis in the case of syndromic cleft palate.

## Background

Orofacial clefts (OFCs), especially lip and/or palate (CL/P cleft), belong to the most prevalent birth defects [1]. Lip and palate development occurs very early in embryogenesis, with the lip forming first followed by the palate [2]. Physiologically, all human embryos have CL and CP that must fuse, however developmental failures at any stage can break necessary fusions and result in OFCs [2]. CL and CP are complex multifactorial disorders, where genetic and/or environmental factors can be involved [3]. Partial autosomal deletions and duplications occur in approximately 1/7000 live birth [4]. Here we describe a further case of a de novo 20p12.3 microdeletion admitted for failure to thrive, psychomotor delay, dysmorphic features and cleft palate. Conventional karyotype displayed a derivative 20 chromosome with an abnormally short p arm. FISH analysis found a de novo 20p12.3 deletion. We report a rare etiology of syndromic cleft palate and we highlight the importance of molecular cytogenetics in diagnosis.

## Case presentation

The proband was referred for chromosome analysis when he was 5 years old because of psychomotor delay and unusual facies. He was the latest child of unrelated, healthy parents. The father was 38 and the mother 28 at the time of his birth. He was born after an uncomplicated 39 weeks pregnancy, and normal vaginal delivery. As a newborn the patient had a short period of cyanosis, and showed congenital hypotonia. Weight was 2100 g (<3rd centile), length 45 cm (<3rd centile), head circumference 34 cm (30th centile). In subsequent evaluation, he showed a failure to thrive and slight delay in the acquisitions of motor milestones; the patient was able to sit with 12 months, and could walk of 2 years, first words were spoken at 36 months, nevertheless, he still retains language disorders and early intervention speech-language therapy was initiated. Length, weight and head circumference at 5 years were 99 cm (<3rd centile), 16 kg (15th), and 50 cm (40th centile), respectively. He had submucous CP with bifid uvula, small forehead, hypertelorism and downslanting palpebral fissures. The nasal bridge was broad and the nasal tip was bulbous. He had low set ears, short philtrum, down turned corners of the mouth and micrognathia. The remaining physical examination was notable for widespread tooth decay and dental overlapping. There were no significant limb anomalies, or cardiovascular disorders. CT scan of the brain was normal, electroencephalogram showed no paroxysmal abnormalities. Ophthalmologic examination and thyroid functions were normal. X-ray examination showed that his bone age was 2 years. Family history revealed an older brother with bilateral cleft lip, but further details were not available.

### Cytogenetic and molecular cytogenetic analysis

Cytogenetic analysis was carried out on the patient and his parents. The study included peripheral lymphocyte culture by a standard method using a reverse banding technique (RHG banding), and G-banding technique using trypsin. About 0.4–0.8 mL of peripheral blood was incubated in complete lymphocyte culture medium for 72 h. Metaphases were harvested by adding karyomax colcemid solution for 50 min followed by hypotonic KCl (0.075 M) treatment for 20 min and fixation using standard 3:1 methanol and acetic acid fixative [5]. At least 11 metaphases were scored. A high-resolution analysis was done by synchronization using thymidine solution (15 mg/mL) for 16 h before harvesting [5]. Fluorescence in situ hybridization (FISH) was performed on patient’s metaphases obtained from whole blood cultures. Subsequent probes were applied on lymphocyte metaphase spreads prepared from the propositus: centromere 20 specific probe (D20Z1 in 20q11.1, Abbott/Vysis, Wiesbaden, Germany), whole chromosome paint for chromosome 20 (homemade WCP), bacterial artificial chromosome (BAC) clones; RP11-96L6 in 20p11.21, and RP11-116E13 in 20p12.3 [6].

## Results

**Fig. 1:**
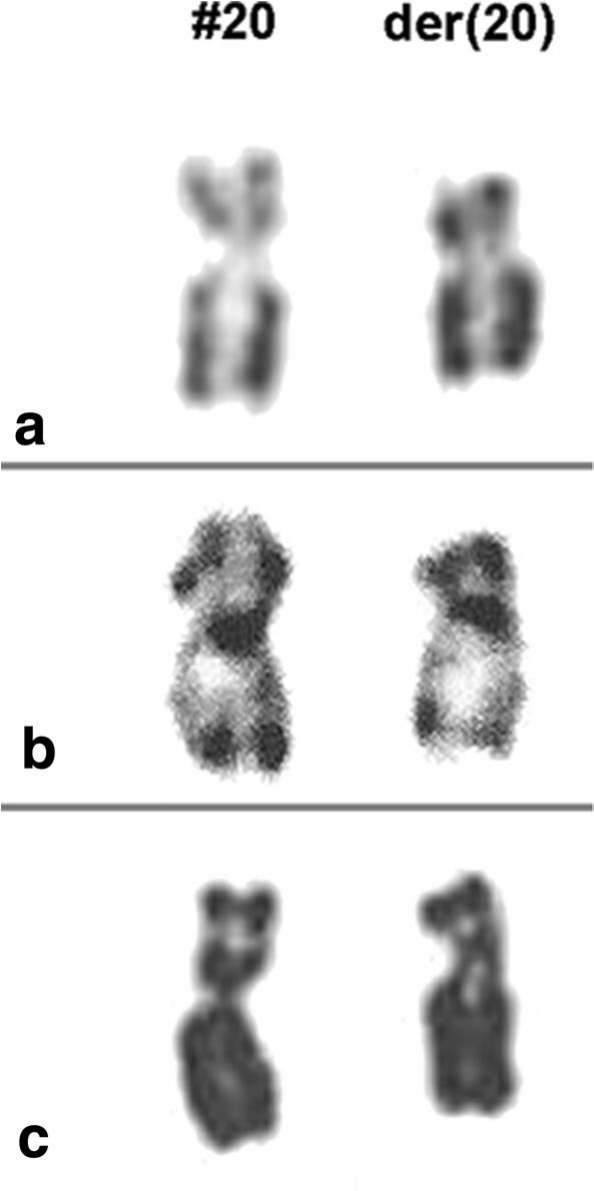
RHG (**a**), GTG banded (**b**) and high resolution partial karyogram (**c**) show a shortened p-arm in chromosome 20

**Fig. 2:**
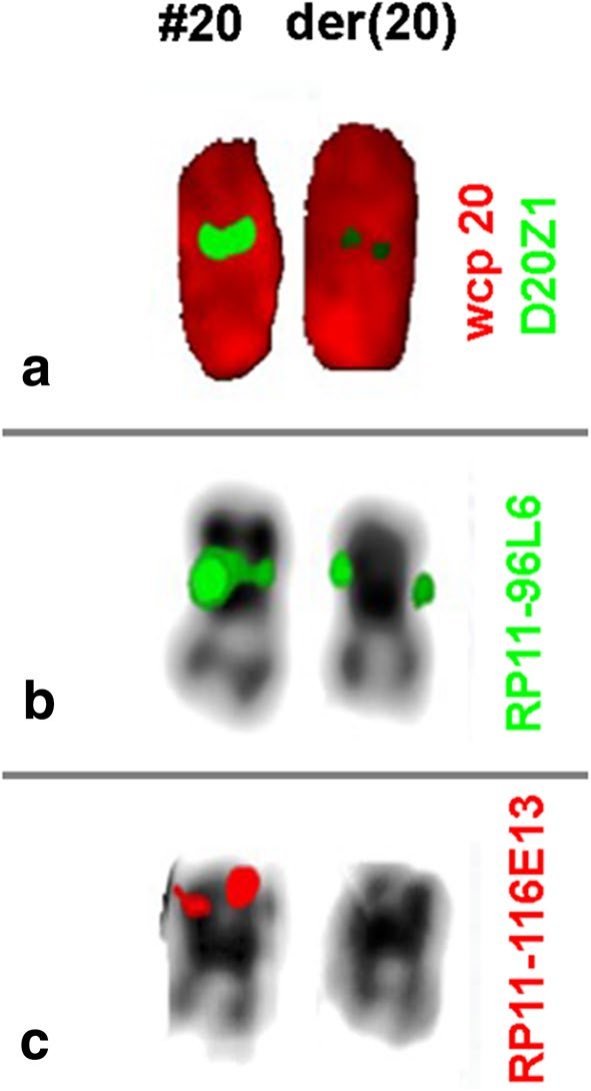
**a** FISH result after application of WCP 20 and CEP 20 with revealing a 20 cen- on der(20). **b** BAC RP-96L6 excluded a deletion in 20p11.21. **c** BAC clone RP11-116E13 confirmed the presence of a deletion in 20p12.3

RHG-banding and high resolution analysis showed a notably short p arm of one chromosome 20 (Fig. 1). FISH analysis identified a 20p12.3 deletion (Fig. 2b, c). With a much smaller D20Z1 positive region than usually observed (Fig. 2a). Since parental karyotypes were both normal, the final karyotype was designated as follows: 46, XY, del(20)(p12.3p12.3), 20cen-dn (de novo). Cytogenetic analysis in elder brother was not available.

## Discussion

Interstitial deletions of the short arm of chromosome 20 are reported in less than 60 cases [7]. To the best of our knowledge only three reports described 20p12.3 deletions like the presently reported patient. Table 1 shows clinical finding of patients reported in the literature with 20p12.3 deletions. Our proband displayed some typical features, most notably failure to thrive, characteristic facial appearance (hypertelorism, downslanting palpebral fissures, broad nasal bridge, bulbous nose, microstomia, and micrognathia); but he lacked digital anomalies and heart defects. Only our patient had widespread tooth decay and dental overlapping that is an uncommon finding and never reported. Cleft palate was inconstant in patients reported above [8]. Ventricular preexcitation and cognitive delay has been reported once [8].

**Table 1:** Clinical features in patients with 20p12.3 deletion

First author of reference		Deletion	Congenital abnormalities
Our patient		De novo 20p12.3 deletion	Failure to thrive, psychomotor delay Small forehead, hypertelorism, downslanting palpebral fissures, low set ears, broad nasal bridge, bulbous nose, short philtrum, down turned corners of the mouth, microstomia, micrognathia, widespread tooth decay, dental overlapping Cleft palate, bifid uvula
Lalani et al. [8]	Patient 1	De novo 20p12.3 deletion	*Failure to thrive*, Neurocognitive delay *Downslanting palpebral fissures*, bilateral epicanthal folds, *broad nasal root and bridge*, malar hypoplasia, full cheeks*, microstomia*, Persistent fetal pads Wolff parkinson white syndrome
	Patient 2	Maternal 20p12.3 deletion	*Short stature*, Macrocephaly, Neurocognitive delay, *motor delay* Frontal upsweep, *downslanting palpebral fissures*, epicanthal folds, *hypertelorism*, small ears with thickened helices, long philtrum, *microstomia*, Broad thumbs/toes with persistent fetal pads Wolff parkinson white syndrome
	Patient 3	De novo 20p12.3 deletion	Macrocephaly, Neurocognitive delay, *motor delay* *Hypertelorism*, malar hypoplasia, persistent fetal pads
Sahoo et al. [9]	Patient 1	Maternal 20p12.3 deletion	*Failure to thrive, microcephaly micrognathia*, Pierre Robin sequence, large communicating fontanelles, long philtrum, pectus excavatum, diastasis recti, gap between halluces and second toes, vertical creases, deep palmar flexion creases, short 5th fingers *Cleft palate* Patent foramen ovale
	Patient 2	?	*Failure to thrive, microcephaly, language deficits* Large eyes, synophrys, long philtrum, upturned nose, *microstomia* Deafness, cholesteatoma *Cleft palate*, high-arched hard palate, *bifid uvula* Zygodactylous triradius between the 2nd and 3rd rightoes
	Patient 3	De novo 20p12.3 deletion	*Failure to thrive, motor deficits* with a head lag and wobble, delayed/poor reflexes, central hypotonia and feeding difficulties Flat facial profile, prominent forehead, *downslanting palpebral fissures*, depressed nasal bridge, small, upturned nose with anteverted nares, pinpoint hemangioma on the tip of the nose, long philtrum, *micrognathia*, transverse crease across the chin Decreased muscle mass, subtle hyperextensibility of hands and feet, recurrent hip dislocations and developmental dysplasia of the hips U-shaped *cleft palate*
Williams et al. [7]		Paternal 20p12.3 deletion	*Failure to thrive, microcephaly, psychomotor delay, microretrognathia*, widened palpebral fissures, long philtrum, digital hypoplasia of second and third toes, self-stimulatory behaviors, mild dextroscoliosis, central incisors, bifid uvula, *cleft palate*

Words in italics represents features in common with our patient

In the present case, FISH analysis allowed us to identify 20 chromosome derivative as a de novo 20p12.3 deletion. This variant was the most predominant one, reported previously in three other patients [8, 9]. However, inherited forms were also described, often maternal [8, 9], and scarcely paternal [10]. Based to the NCBI map viewer (http://www.ncbi.nlm.nih.gov/projects/mapview/), 11 annotated genes within 20p12.3 region were found, notably *BMP2* gene. This gene is well known to play a critical role in bone formation, as well as being implicated in a wide range of functions in morphogenesis, including palate morphogenesis. Moreover, the widespread expression of *BMP2* in cells of skeletal, neurological, cardiac, and other tissues supports the pleiotropic effects of *BMP2* haploinsufficiency. This gene is also expressed in post natal odontoblast and ameloblast during tooth differentiation. Interestingly, its deletion in early odontoblast results in a permanent tooth phenotype [11]; this may explain dental abnormalities in our patient, but further cases with similar microdeletion and mutational analysis of *BMP2* are required to delineate this genotype–phenotype correlation.

## Conclusion

We describe here a Moroccan patient with psychomotor delay and facial dysmorphism, in which a de novo 20p12.3 deletion was identified, and we highlight the interest of FISH in the diagnostic confirmation of chromosomal rearrangement. In practice, 20p12.3 deletion can be considered as an etiological diagnosis of syndromic cleft palate.

## Consent

Written informed consent was obtained from the patient’s parents for publication without image of this case report.

## Abbreviations

OFC: orofacial cleft
CL: cleft lip
CP: cleft palate
FISH: fluorescent in situ hybridization
